# Mechanism of Liver Regeneration During ALPPS

**DOI:** 10.3389/fcell.2022.916286

**Published:** 2022-06-08

**Authors:** Yao Xiao, Lantao Peng, Hongjuan Xu, Ming Huang, Chao Yang, Guodong Liu, Xiwu Ouyang, Xiaoli Li, Yuanjing Wang, Langqing Sheng, Denggao Zhai, Ling Lin, Ling Liu, Gang Liu, Liansheng Gong

**Affiliations:** ^1^ Department of General Surgery, Xiangya Hospital, Central South University, Changsha, China; ^2^ International Joint Research Center of Minimally Invasive Endoscopic Technology Equipment and Standards, Changsha, China; ^3^ Department of Nuclear Medicine, Key Laboratory of Biological Nanotechnology of National Health Commission, Xiangya Hospital, Central South University, Changsha, China; ^4^ National Clinical Research Center for Geriatric Disorders, Xiangya Hospital, Central South University, Changsha, China; ^5^ Department of General Surgery, Hunan Yueyang Maternal and Child Health-Care Hospital, Yueyang, China; ^6^ Department of General Surgery, The First Hospital of Changsha, Changsha, China; ^7^ Department of Hepatobiliary Surgery, Shanxi Bethune Hospital, Taiyuan, China; ^8^ Department of Geriatric Surgery, Xiangya Hospital, Central South University, Changsha, China

**Keywords:** circRNA, liver regeneration, ALPPS, cell proliferation, liver cancer (LC)

## Abstract

Liver cancer is one of the most lethal malignant tumors in the world, and surgical resection is the main treatment for liver cancer. Liver failure due to insufficient residual liver volume is a fatal complication after hepatectomy. How to effectively increase the residual liver volume after hepatectomy and improve the safety of hepatectomy has always been a problem to be solved in liver surgery. Associating liver partition and portal vein ligation for staged hepatectomy (ALPPS) effectively reduces the occurrence of liver failure due to insufficient residual liver volume after hepatectomy, thereby increasing the probability of radical resection by inducing rapid proliferation of residual liver tissue. However, the molecular mechanism of residual liver tissue regeneration after primary ALPPS (combined liver partition and portal vein ligation) remains unclear. Here, we found that lots of circular RNAs (circRNAs) are upregulated after ALPPS in pig liver cells; then, we identified the orthologous circRNA in humans and pigs to detect their function in liver regeneration. The results showed that loss of circ-0067724 and circ-0016213 could suppress liver cell proliferation. Together, these findings suggest that circ-0067724 and circ-0016213 play an important role in liver cell proliferation, and this may help us to find new strategies to promote liver regeneration.

## Introduction

Primary liver cancer (PLC) is one of the main malignant tumors that seriously endanger human health (Jemal et al., 2011; Bray et al., 2018). Based on the literature reports, the incidence and mortality of PLC are ranked fifth and second, respectively, in the world (Mattiuzzi and Lippi, 2020; Sung et al., 2021). PLC is the second most fatal and fourth most commonly diagnosed malignant tumor in China (Zheng et al., 2019). The American Cancer Society research studies show that there will be more than 40,000 new cases, and more than 30,000 new deaths of PLC occurred in the US in 2021 (Siegel et al., 2021).

The most effective treatment of liver cancer is surgical resection, but its initial resection rate is low (Shabunin et al., 2020). For patients with primary or metastatic liver tumors that are expected to have insufficient future liver remnant (FLR), ALPPS may be considered for radical resection (Yao et al., 2014; Petrowsky et al., 2020). The German scholar Hans Schlitt found that the FLR was insufficient during the operation of a patient with hilar cholangiocarcinoma, so he temporarily decided to carry out a two-step hepatectomy with liver partition and right portal vein ligation in 2007 (Narita et al., 2012). Hauke Lang presented this procedure at the European-African Hepatobiliary and Pancreatic Conference in 2011. Schnitzbauer et al. (2012) published the relevant research results of ALPPS in 2012. This research result not only excites people but also raises many doubts. Reports on ALPPS increased rapidly after the 2015 ALPPS conference, which was held in Hamburg, Germany. Simultaneous portal and hepatic vein embolization (PVE/HVE) holds promise to induce accelerated liver regeneration in a similar safety profile to portal vein embolization (PVE). The demonstrated accelerated hypertrophy might increase the resectability. Randomized trials will have to compare PVE/HVE and PVE to determine if PVE/HVE is superior to PVE (Heil and Schadde, 2021).

Circular RNA (circRNA) is a popular and special type of non-coding RNA molecule. The circRNA molecule has more stable expression than traditional linear RNA, and it is not easily degraded (Memczak et al., 2013). CircRNA has been well documented to play important roles in the regulation of cellular processes (Xiao et al., 2020). CircRNA works through microRNAs (miRNAs), and miRNAs are short in length (20–24 nt), which are major posttranscriptional regulators of gene expression. They form base pairs with the complementary target mRNA at the 3′UTR and modulate cellular processes by repressing the mRNA translation or degrading the mRNA (Salim et al., 2022).

Here, we found that loss of circ-0067724 and circ-0016213 can suppress liver cell proliferation. Together, these finding suggest that circ-0067724 and circ-0016213 play an important role in liver cell proliferation, and this may help us find new strategies to promote liver regeneration.

## Materials and Methods

### Patient Section

The design of this study was approved by the Ethics Committee of XiangYa Hospital. All clinical data involved in this study were obtained with patient consent. A total of 12 liver cancer patients were included from January 2015 to December 2019. The patients with primary or metastatic liver tumors did not have insufficient future liver remnant (FLR). All patients underwent associating liver partition and portal vein ligation for staged hepatectomy and were diagnosed with primary (*n* = 8) or metastatic (*n* = 4) liver cancer by postoperative pathology. Patients with infectious diseases, autoimmune diseases, pregnancy, and liver cancer patients who previously underwent transarterial chemoembolization (TACE) or other treatments, and patients with incomplete data were excluded.

### Animal Studies

Pigs were randomly divided into the control group, liver partition group, portal vein ligation group, and ALPPS group, and six pigs were included in each group. All pigs were preoperatively fasted for 12 h and were fixed on the operating table after anesthesia. Ventral midline incision was around 10 cm long. We exposed the liver and hepatic portal vessels. 1) Control group: the abdominal cavity was disturbed without portal vein ligation and liver separation. 2) Liver partition group: the liver was separated with an electric knife. 3) Portal vein ligation group: the portal vein was separated, and the right branch of the portal vein was ligated. 4) ALPPS: the portal vein was separated, and the right branch of the portal vein was ligated. The ischemic line was seen in the middle lobe of the liver, and then, the ischemic line was separated with an electric knife (without damaging the hepatic portal vessels). The bleeding was stopped, and the abdomen was closed. The pigs grow freely. Animal experiments were approved by the Institutional Animal Care and Use Committee at the Xiangya Hospital of Central South University.

### Residual Liver Volume Measurement

The liver volume was calculated using a CT scan (for human) or drainage method (for animal). Pigs were sacrificed after 2 weeks; the left liver was placed in a measuring cylinder filled with water, and the elevated volume of liquid represented the residual liver volume.

### RNA Isolation and Library Preparation

For RNA extraction, the TRIzol reagent (Invitrogen) was used. RNA purity and quantification were evaluated using the NanoDrop 2000 spectrophotometer (Thermo Fisher Scientific, Waltham, MA, United States). RNA integrity was assessed using the Agilent 2100 Bioanalyzer (Agilent Technologies, Santa Clara, CA, United States).

### Cell Culture

The human hepatocyte HL-7702 cell line was purchased from the Cell Bank of the Chinese Academy of Sciences (Shanghai, China) and cultured in Dulbecco’s modified Eagle’s medium (DMEM, Invitrogen), with 10% FBS, 1% penicillin/streptomycin, and 1% glutamine. All cells were cultured in 5 percent (v/v) carbon dioxide in a humidified incubator at 37°C.

### MTT Assay

The MTT assay was used to detect cell proliferation rates. Cells were transfected with plasmids for 24 h, and then seeded in 96-well plates at 5,000 per well. On day 2, we added the MTT reagent to each well and then incubated the plates at 37°C. After 2 h, we dissolved the precipitate in DMSO and measured the absorbance at 450 nm. Each sample was assayed in triplicate.

### qRT-PCR Assay

The TRIzol reagent (Invitrogen) was used to extract the total RNA of cells, and 2 μg of total RNA was used for the reverse transcription. The Bio-Rad CFX96 system was used to conduct and calculate the expression of RNA. The data were normalized by GAPDH, and the relative expression was assessed by 2^−ΔΔCT^ values. All primers were purchased from Integrated DNA Technologies, Inc. circ-0067724, F: TTT​GTC​CAG​GAT​AGA​CAT​AGA​GC and R: ATG​GGT​TCA​CAG​GCA​TTC​TC; circ-0016213, F: TGG​CAG​TTC​GAA​AAA​GAA​AAA and R: GGC​CCG​AAT​CTC​TTC​CAT​A.

### Plasmid Construction

In the pLKO.1-shcircRNA plasmids, HL-7702 cells were transfected using the Lipofectamine 3000 transfection reagent (Invitrogen, Carlsbad, CA), according to the manufacturer’s instructions. The pLKO.1-shcircRNA construct sequences are shown in Supplementary Data.

### Statistical Analysis

All statistical analyses were carried out with SPSS 19.0 (SPSS Inc., Chicago, IL). All experiments were run with samples in triplicate and at least three separate times. The data values were presented as the mean ± SD. Differences in mean values between two groups were analyzed by two-tailed Student’s *t*-test. The p value < 0.05 was considered statistically significant.

## Results

### ALPPS Could Promote Human Liver Regeneration

**FIGURE 1:**
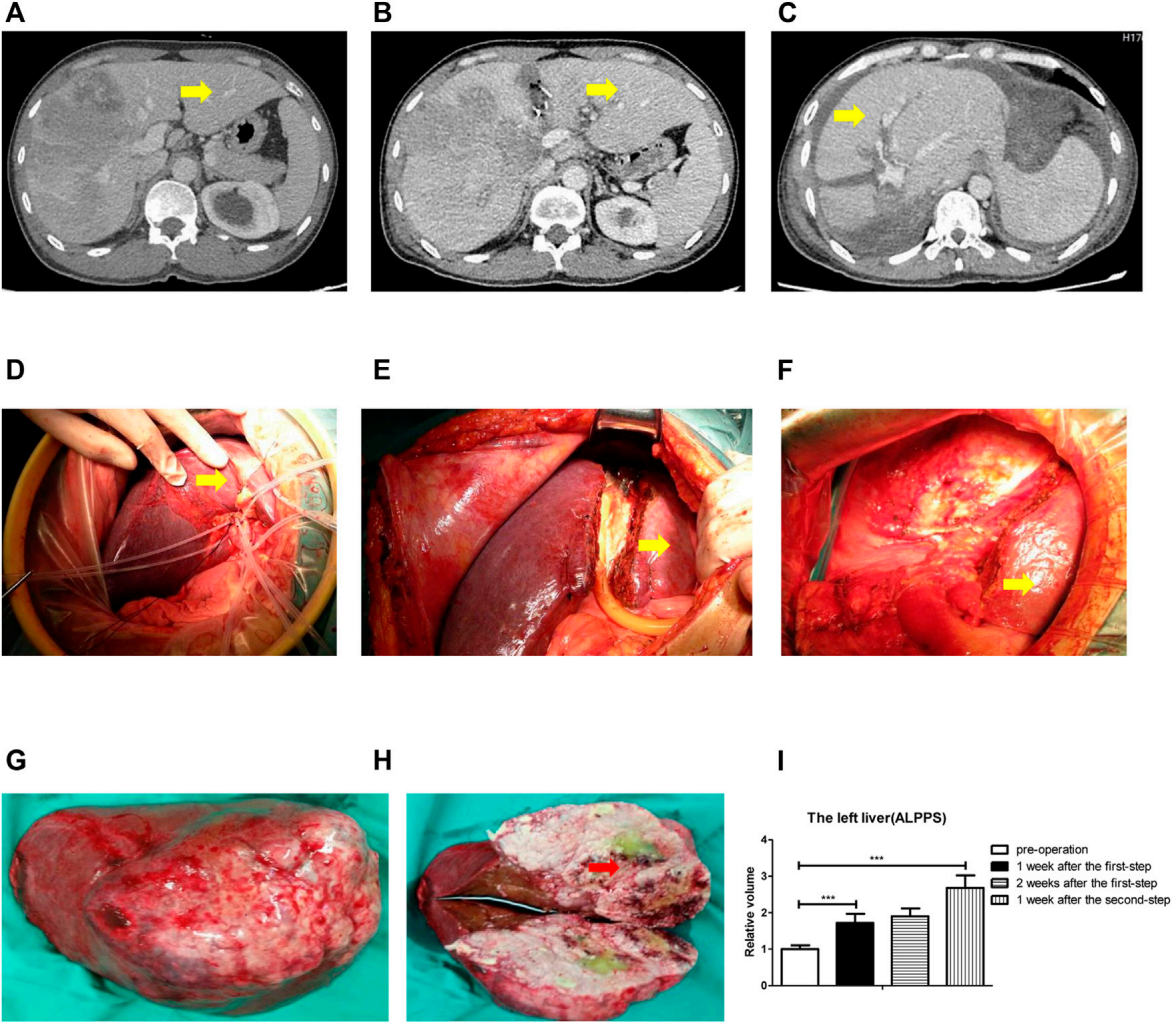
ALPPS could promote human liver regeneration. **(A)** CT scan showed the liver before surgery. **(B)** CT scan showed the liver 2 weeks after the first-step surgery. **(C)** CT scan showed the liver 1 week after the second-step surgery. **(D)** Right branch of the portal vein was ligated. **(E)** Ischemic line was separated. **(F)** Right hepatectomy on the patient. **(G)** Right hepatic tumor was completely resected. **(H)** Tumor was atrophic with ischemic necrosis. **(I)** Calculation of the left liver relative volume with a CT scan at four different time points: before operation, 1 week after the first-step operation, 2 weeks after the first-step operation, and 1 week after the second-step operation. The quantification is presented as mean ± SD, ***p < 0.001.

ALPPS is an innovative technique in liver surgery in recent years. In our study, we first operated ALPPS on liver cancer patients. All patients underwent CT examination to calculate the volume of the liver; the yellow arrow indicates the left liver at three different time points: before operation (Figure 1A), 2 weeks after the first-step operation (Figure 1B), and 1 week after the second-step operation (Figure 1C). The CT scan showed (Figures 1A–C) a significant increase in left liver volume (yellow arrow). During operation, we first separated the portal vein, and the right branch of the portal vein was ligated (Figure 1D). The ischemic line was seen in the middle lobe of the liver, and then, the ischemic line was separated with an electric knife (without damaging the hepatic portal vessels) (Figure 1E). Two weeks later, the left liver (yellow arrow) was significantly enlarged (Figure 1F), so we performed a right hepatectomy on the patient. The right hepatic tumor was completely resected (Figure 1G), and the tumor was atrophic with ischemic necrosis (red arrow) (Figure 1H). The left liver grew rapidly in 2 weeks postoperatively (Figure 1I).

Together, the data from Figure 1 indicated that ALPPS could promote human liver regeneration.

### ALPPS Could Promote Pig Liver Regeneration

**FIGURE 2:**
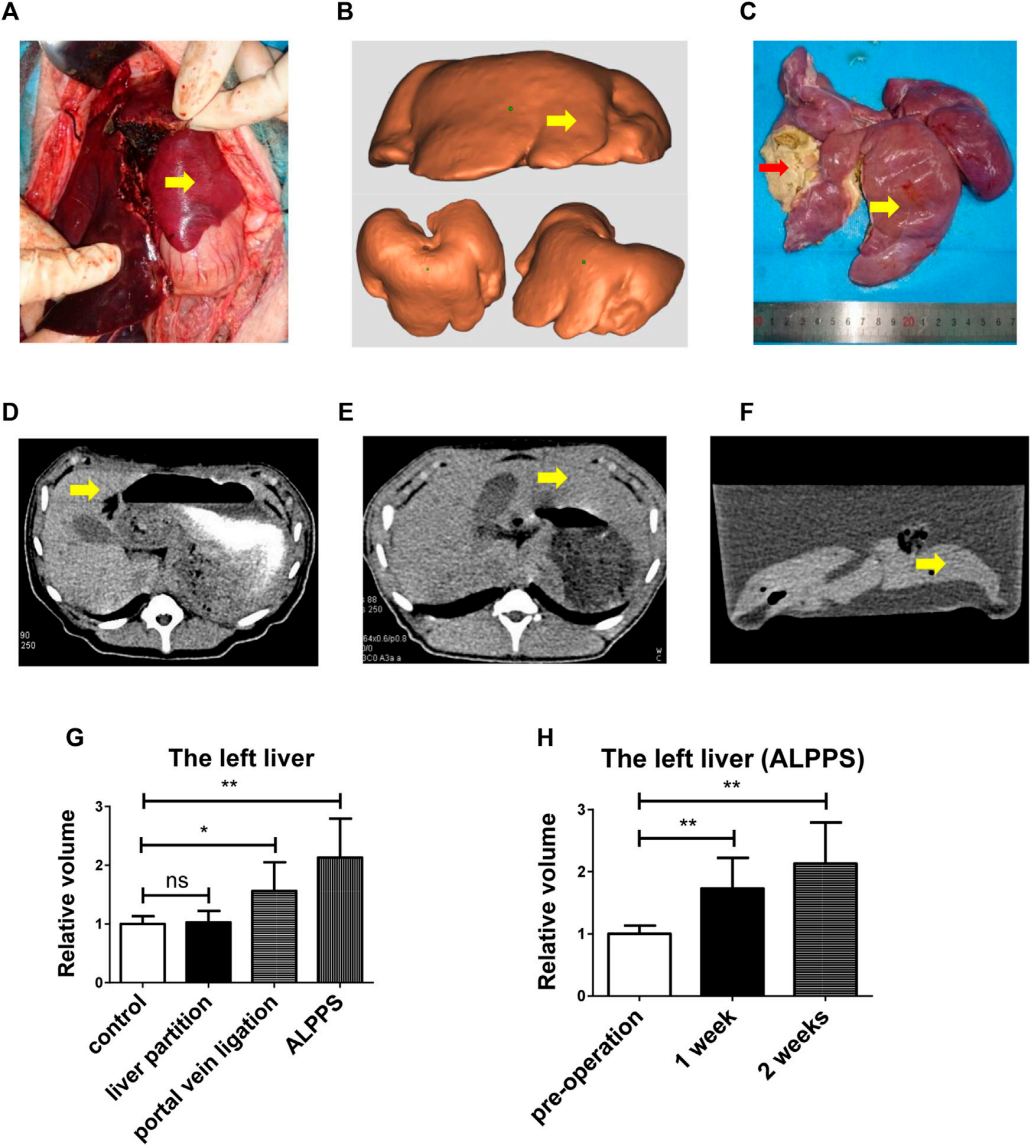
ALPPS could promote pig liver regeneration. **(A)** ALPPS operation on pigs, and the yellow arrow indicates the left liver. **(B)** 3D-printed model of pig liver. **(C)** Pigs were sacrificed after 2 weeks; the left liver (yellow arrow) and the right liver (red arrow). **(D)** CT scan showed the liver before surgery. **(E)** CT scan showed the liver 2 weeks later (before sacrificing). **(F)** CT scan showed the liver 2 weeks later (after sacrificing, liver in water). **(G)** Left liver relative volume in four groups (ctrl, liver partition, portal vein ligation, and ALPPS). **(H)** Calculation of the left liver relative volume with a CT scan. The quantification is presented as mean ± SD, *p < 0.05, **p < 0.01.

ALPPS could promote human liver regeneration (Tomassini et al., 2019), but the mechanism of its rapid and stable promotion of FLR regeneration in the short term remains unclear. Then, we operated ALPPS on pigs (Figure 2A), and the yellow arrow indicates the left liver. We took a CT scan of the pig to reconstruct the liver and 3D-printed it (Figure 2B). The left liver (yellow arrow) was significantly enlarged (Figure 2C), and the right liver was atrophic with ischemic necrosis (red arrow). The CT scan showed the liver before surgery (Figure 2D), and 2 weeks later, the CT scan showed (Figures 2E,F) a significant increase in left liver volume (yellow arrow). The left liver increased more significantly in the ALPPS group than the portal vein ligation group; however, there was no change in the liver partition group contrast to the control group (Figure 2G). The left liver grew rapidly postoperatively (Figure 2H).

Together, the data from Figure 2 indicated that ALPPS could promote pig liver regeneration.

### High-Throughput Sequencing for FLR

**FIGURE 3:**
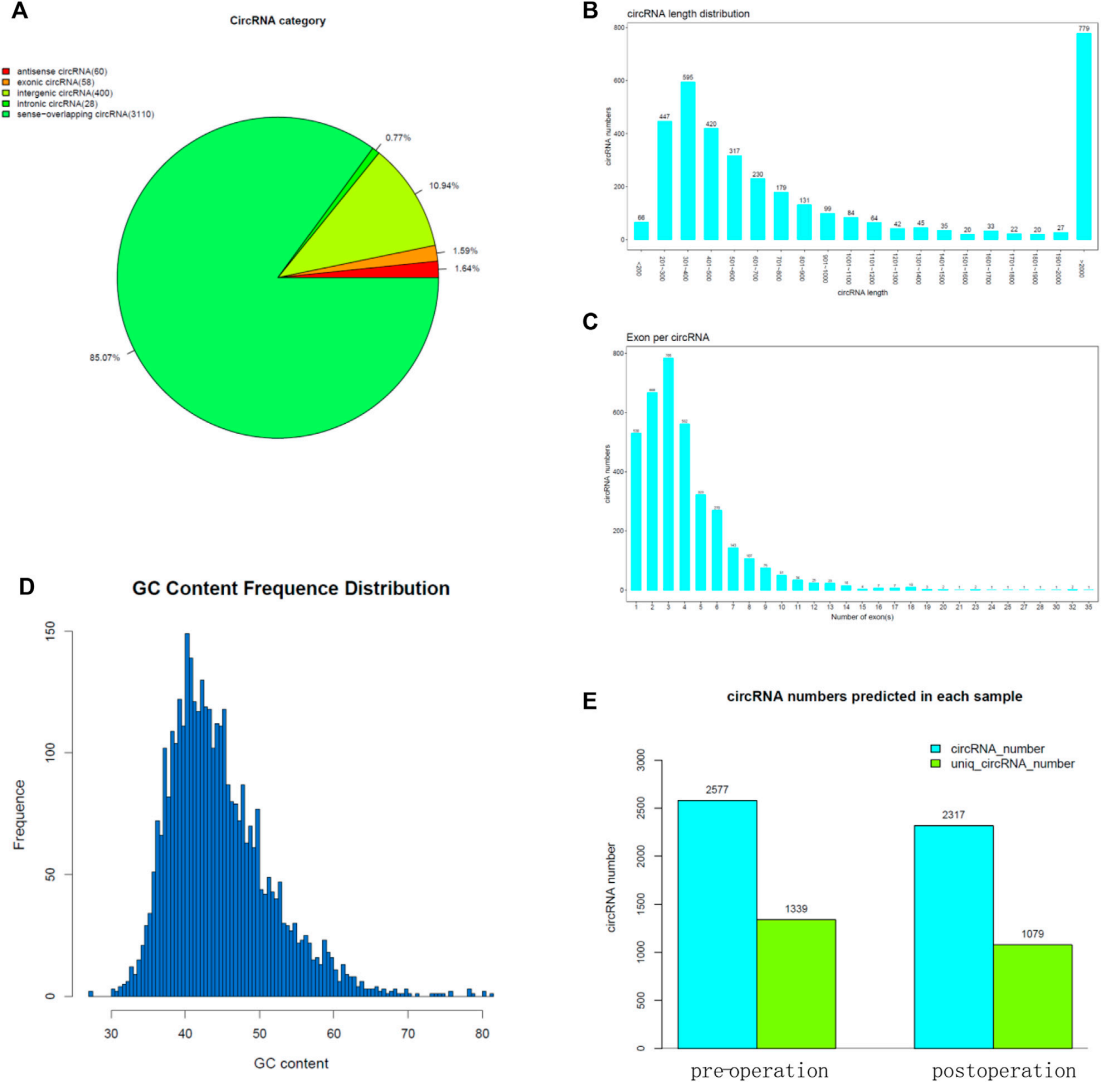
High-throughput sequencing for FLR. **(A)** CircRNA category of the high-throughput sequencing result. **(B)** CircRNA length of the high-throughput sequencing result. **(C)** Exon number per circRNA. **(D)** GC content frequency distribution of circRNA. **(E)** CircRNA numbers predicted in each sample.

CircRNAs have been well documented to play important roles in promoting cancer progression. We collected preoperative and postoperative FLR tissue from pigs for high-throughput sequencing. The result showed that the circRNA category include antisense circRNA, exonic circRNA, intergenic circRNA, intronic circRNA, and sense-overlapping circRNA (Figure 3A). Most of the circRNA length is 201–700 bp (Figure 3B), and some of them are more than 2 kb. Almost all of them comprise less than 10 exons (Figure 3C). The GC content frequency distribution of the most circRNA is 35–55% (Figure 3D). The result showed the circRNA numbers predicted in each sample (Figure 3E).

### Loss of circ-0067724 or circ-0016213 Could Suppress Liver Regeneration

**FIGURE 4:**
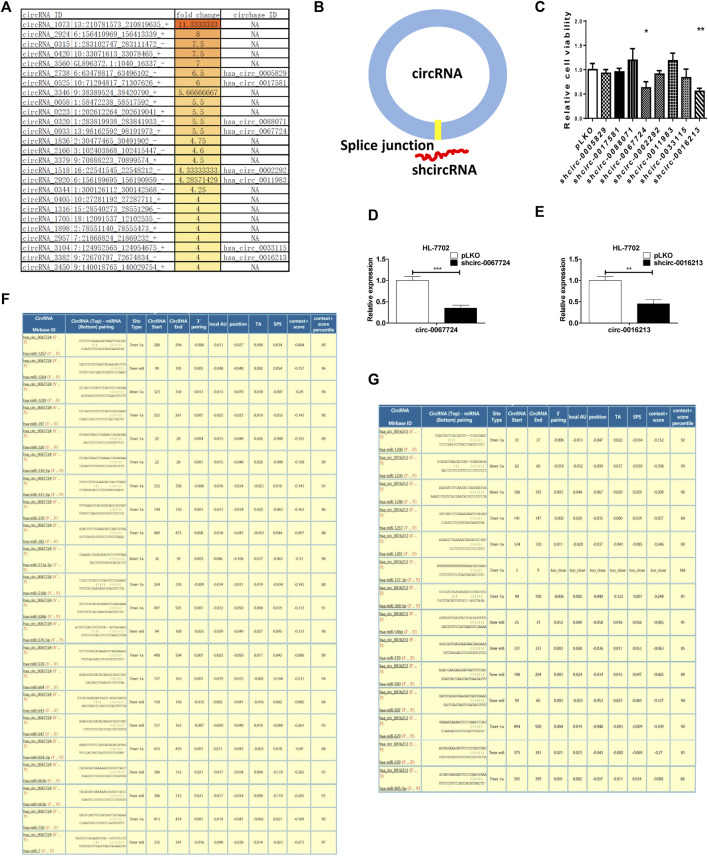
Loss of circ-0067724 or circ-0016213 could suppress liver regeneration. **(A)** Top 26 increased circRNAs in postoperative left liver tissue and eight homologous genes of humans and pigs. **(B)** Schematic illustration showing the position of the targeting shRNA to knock down the circRNAs. **(C)** MTT assays to detect the function of shcircRNA. **(D,E)** qRT-PCR was used to confirm the knock down efficacy in HL-7702. **(F,G)** Potential downstream miRNAs relative to circ-0067724 or circ-0016213. The quantification is presented as mean ± SD, *p < 0.05, **p < 0.01, and ***p < 0.001.

We focused on increased circRNAs in postoperative left liver tissue and selected 26 increased circRNAs with the greatest variability (Figure 4A); however, these were pig circRNAs but not human circRNAs. By homology comparison of the circRNAs and human circRNA sequence, we found eight of them are highly homologous. To further examine whether the eight circRNAs promote liver regeneration, we constructed shRNAs for these eight circRNAs (sh-circRNA) by targeting specific splice junctions (Figure 4B). The results from the MTT assays revealed that only knocking down circ-0067724 and circ-0016213, but not the other six, can suppress liver cell proliferation (Figure 4C). We used qRT-PCR to confirm the knock down efficacy in HL-7702 (Figures 4D,E). Based on the MTT results, we predicted the potential downstream miRNAs relative to circ-0067724 (Figure 4F) or circ-0016213 (Figure 4G) by Circular RNA Interactome (https://circinteractome.nia.nih.gov/index.html).

## Discussion

Primary liver cancer (PLC) is one of the most important public health problems facing the world. The incidence of HCC in the United States has tripled in the past 30 years (El-Serag, 2011).

Hepatectomy is one of the most important treatment methods for liver surgery diseases, especially for primary or metastatic liver malignant tumor diseases; surgical resection is not only the preferred treatment method but also the only possible to achieve a cure (Sucandy et al., 2020). In the past 30 years, with the development of liver surgery, improvement of surgical techniques, and standardization of intraoperative and perioperative management, the restricted area of hepatectomy has been continuously broken through, and the success rate of operation has been significantly improved; in addition, the incidence of postoperative complications and mortality have decreased significantly (Wei Chieh et al., 2020). However, an important factor restricting hepatectomy is insufficient FLR. Postoperative liver failure caused by insufficient FLR is a fatal complication after hepatectomy and one of the common causes of death after extensive hepatectomy. Therefore, how to increase the volume of FLR before surgery and improve the safety of hepatectomy has always been a problem which liver surgery is committed to solve.

The appearance of ALPPS has caused an unprecedented sensation in the field of hepatobiliary surgery. As a new technique of staged hepatectomy, ALPPS will play an important role in promoting the development of hepatobiliary surgery because it is expected to solve the bottleneck problem of hepatectomy, which is insufficient residual liver volume (Fiorentini et al., 2021; Hasselgren et al., 2021). Because ALPPS available time is shorter, the number of cases of clinical implementation is limited; some problems related to ALPPS have yet to be further clarified, such as the mechanism of significant liver regeneration caused by the joint orthotopic liver partition is unclear and how to reduce the high postoperative complications’ incidence and mortality rate. Our research focused on the role of circRNAs in liver regeneration.

CircRNAs have been proven to be widely present in many organs, which are a class of long non-coding RNA molecules, and circRNAs shape a covalently closed continuous loop which have no 5′-3′ polarity and contain no polyA tail. CircRNA mainly exists in the cytoplasm or exosomes and has the characteristics of tissue specificity, disease specificity, timing specificity, and high stability (Li et al., 2015). In recent years, a large number of studies have shown that circRNA is closely related to stress response, biological growth and development, and disease occurrence and development, but the specific biological function remains unclear. With the development of research, more and more biological functions of circRNA are recognized (Rong et al., 2017). The miRNAs sponge was the most reported. In our study, we found that eight circRNAs increased after ALPPS, and we predicted the potential downstream miRNAs relative to circ-0067724 or circ-0016213 by Circular RNA Interactome. The two circRNAs may act as miRNA sponges to regulating the expression of downstream target genes of miRNAs. Our study was another example to show the biological functions of circ-0067724 and circ-0016213, which can promote liver regeneration, but the specific mechanisms still need further research.

## Data Availability

The datasets presented in this study can be found in online repositories. The names of the repository/repositories and accession number(s) can be found at: https://www.ncbi.nlm.nih.gov/, SUB10127076.
