# Supplementary material for: Mechanism of Liver Regeneration During ALPPS
Source: Front Cell Dev Biol. 2022 Jun 8;10:916286. doi: 10.3389/fcell.2022.916286 (PMC9213876; doi:10.3389/fcell.2022.916286)
Supplement: Supplementary file 1 [file DataSheet1.pdf]

The plasmids sequences used were as follows:

shcirc-0067724:

Forward:

CCGGAAGAAAGGGTTTTTTGATTAATTGGATCCGTTAATCAAAAAACCCTTTCTTTTTTTG

Reverse:

AATTCAAAAAAAGAAAGGGTTTTTTGATTAACGGATCCAATTAATCAAAAAACCCTTTCTT

shcirc-0016213:

Forward:

CCGGGGCTACAGAAATAGGAAGGTTTTGGATCCGAACCTTCCTATTTCTGTAGCCTTTTTG

Reverse:

AATTCAAAAAGGCTACAGAAATAGGAAGGTTCGGATCCAAAACCTTCCTATTTCTGTAGCC
